# 1208. Polymorphic Locus SerMT1 for Sequence-based Typing of *Serratia marcescens*

**DOI:** 10.1093/ofid/ofac492.1041

**Published:** 2022-12-15

**Authors:** Tom Edlind

**Affiliations:** MicrobiType LLC, Wyndmoor, Pennsylvania

## Abstract

**Background:**

Outbreaks of pathogenic agents within healthcare facilities may have sporadic or nosocomial sources. Effective intervention requires distinguishing between these sources, which in turn requires epidemiological investigation in conjunction with typing of pathogen isolates. For the latter, established methods range from ribotyping and pulse-field gel electrophoresis to multilocus sequence typing and whole genome sequencing. Due to technical complexities and costs associated with these methods, however, typing is rarely pursued. Polymorphic locus sequence typing (PLST) addresses these issue by employing conventional PCR and Sanger sequencing; the key to PLST is its focus on a tandem repeat-containing locus exhibiting maximal variation due to combinations of single nucleotide polymorphisms and insertions/deletions. *Serratia marcescens* has been implicated in numerous nosocomial outbreaks, and relatedly has the ability to colonize the hospital environment (e.g., sinks), although few studies have definitively established an outbreak-environment link.

**Methods:**

To extend PLST to *S. marcescens*, tandem repeats were bioinformatically identified in the genome sequence of strain WW4 (accession CP003959) and used with flanking sequences as queries in BLASTN searches of the GenBank nr/nt *Serratia* genome database including 97 *S. marcescens* strains. One locus designated SerMT1 (bp 243212-244195) was present in all strains and exhibited high variability associated with 6-16 copies of a PVVEPE-encoding repeat within signal recognition particle receptor gene *ftsY*.

**Results:**

SerMT1 was downloaded from all 97 strains, aligned using Clustal Omega, and phylogenetically analyzed using DNA parsimony. This analysis resolved 91 total alleles and yielded an impressively high Simpson’s Diversity Index of 0.998. Six clusters of 2 to 4 strains shared SerMT1 sequence; their epidemiologic relatedness was supported by their GenBank annotations (e.g., UMH-10 and UMH-11 isolated the same month at the same facility).

**Conclusion:**

SerMT1 PLST provides an affordable, user-friendly new tool for epidemiologic investigation of *S. marcescens* outbreaks.

**Disclosures:**

**Tom Edlind, PhD**, MicrobiType LLC: Ownership Interest.

